# Dopamine Alters the Fidelity of Working Memory Representations according to Attentional Demands

**DOI:** 10.1162/jocn_a_01073

**Published:** 2016-11-29

**Authors:** Sean James Fallon, Nahid Zokaei, Agnes Norbury, Sanjay G. Manohar, Masud Husain

**Affiliations:** 1 University of Oxford, Oxford, UK; 2 University of Cambridge, Oxford, UK; 3 John Radcliffe Hospital, Oxford, UK

## Abstract

Capacity limitations in working memory (WM) necessitate the need to effectively control its contents. Here, we examined the effect of cabergoline, a dopamine D_2_ receptor agonist, on WM using a continuous report paradigm that allowed us to assess the fidelity with which items are stored. We assessed recall performance under three different gating conditions: remembering only one item, being cued to remember one target among distractors, and having to remember all items. Cabergoline had differential effects on recall performance according to whether distractors had to be ignored and whether mnemonic resources could be deployed exclusively to the target. Compared with placebo, cabergoline improved mnemonic performance when there were no distractors but significantly reduced performance when distractors were presented in a precue condition. No significant difference in performance was observed under cabergoline when all items had to be remembered. By applying a stochastic model of response selection, we established that the causes of drug-induced changes in performance were due to changes in the precision with which items were stored in WM. However, there was no change in the extent to which distractors were mistaken for targets. Thus, D_2_ agonism causes changes in the fidelity of mnemonic representations without altering interference between memoranda.

## Introduction

Working memory (WM), the ability to store and manipulate information in the short term, is a limited capacity system that is essential to our daily lives (Baddeley, 2012; Oberauer & Hein, 2012). Most studies usually assess WM using binary report measures and examine the quantity of information that can be maintained, for example, the number of items that can be recalled. Recent methodological developments also allow us to measure the fidelity or quality of information that can be retained from the latent structure of responding. Although controversial (Ester, Vogel, & Awh, 2012; Zhang & Luck, 2011), it has been proposed that WM might be best understood as a finite resource that can be distributed among retained items, with decrements in recall precision as set size increases (Ma, Husain, & Bays, 2014; Bays, Catalao, & Husain, 2009; Bays & Husain, 2008; Alvarez & Cavanagh, 2004; Wilken & Ma, 2004).

An important inference that has emerged from the use of recall precision measures is that the distribution of resources can be flexibly altered depending on task demands. For example, dynamic reallocation of WM resource has been observed in both precueing and retrocueing experiments: When participants selectively attend to one item among several others, the precision with which they recall it is significantly enhanced (Zokaei, Manohar, Husain, & Feredoes, 2014; Zokaei, Ning, Manohar, Feredoes, & Husain, 2014; Pertzov, Bays, Joseph, & Husain, 2013; Gorgoraptis, Catalao, Bays, & Husain, 2011; Bays & Husain, 2008). Indeed, precueing can modulate the effect distractors have on recall such that performance for the cued item becomes equivalent to the case where no distractors are present (Gorgoraptis et al., 2011), suggesting that top–down mechanisms are highly effective in preventing irrelevant items from gaining mnemonic resources.

The neurotransmitter dopamine is a strong candidate for supporting such reallocation given its long-standing association with WM maintenance (Rypma et al., 2015; Eckart, Fuentemilla, Bauch, & Bunzeck, 2014; Fischer et al., 2010; Vijayraghavan, Wang, Birnbaum, Williams, & Arnsten, 2007; Floresco, Magyar, Ghods-Sharifi, Vexelman, & Maric, 2006; Goldman-Rakic, 1995) and the prominent role attributed to the D_2_ receptor in controlling the contents of WM (Bloemendaal et al., 2015; Cools & D’Esposito, 2011; Hazy, Frank, & O’Reilly, 2007; Mehta, Manes, Magnolfi, Sahakian, & Robbins, 2004). Dopaminergic processing in the striatum may make an essential contribution to WM by modulating cortical processing (Chatham & Badre, 2015). In attention, it has been demonstrated that the BG can modulate activity in the sensory cortex by modulating the connectivity through boosting activity of task-relevant areas and decreasing activity in task-irrelevant areas (van Schouwenburg, den Ouden, & Cools, 2013). More specifically, with regard to WM, a division of labor has been proposed between the D_1_-dominated go and the D_2_ no-go corticostriatal pathways. It has been hypothesized that, whereas the go pathway allows entry of items into WM, the no-go pathway prevents it (Hazy et al., 2007; Frank & O’Reilly, 2006). Thus, D_2_ receptors might be involved in controlling the contents of WM by preventing irrelevant information from gaining access to scarce mnemonic resources. Specifically, under this model, postsynaptic stimulation of D_2_ receptors should inhibit the no-go pathway, inhibiting the gating of information and thus making WM more open to external input.

**Figure 1:**
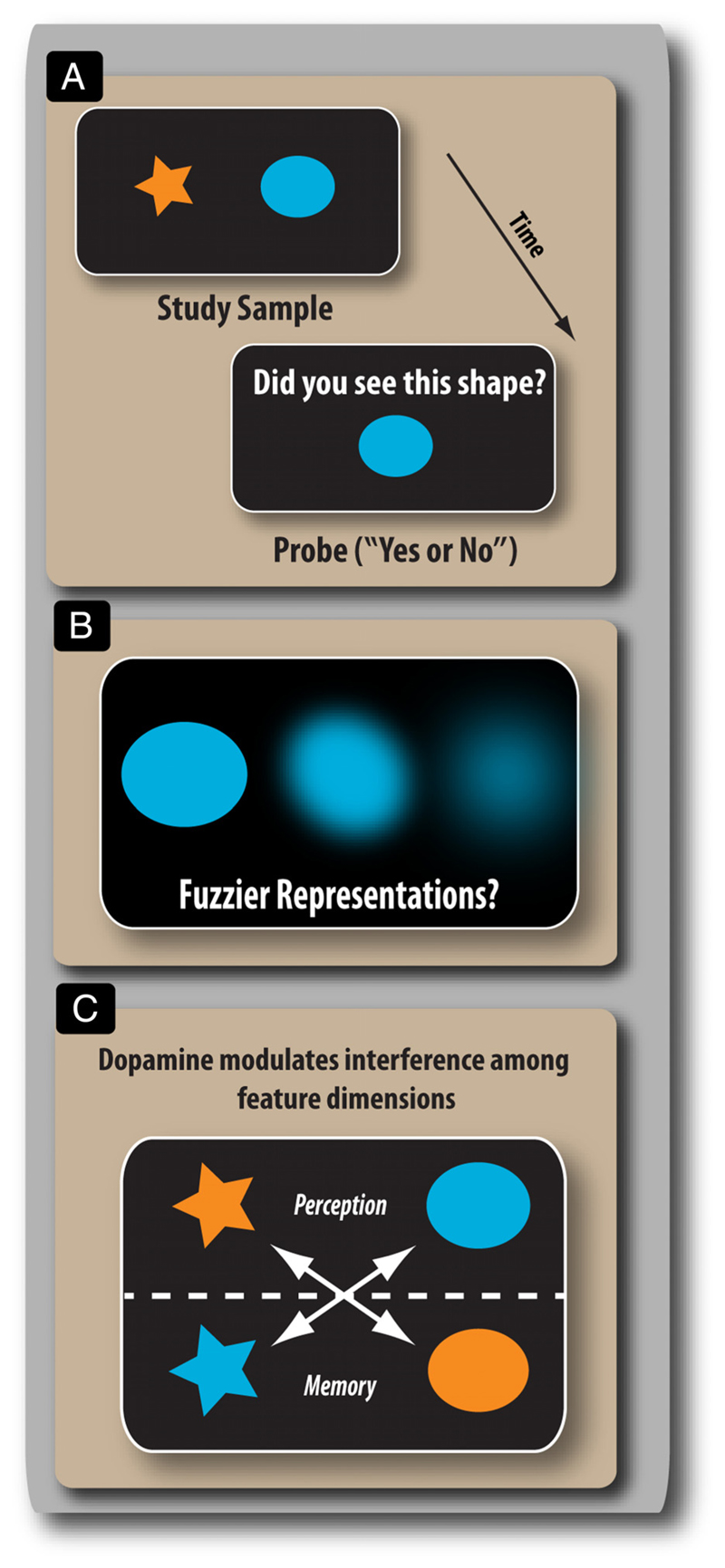
Potential effects of dopamine on WM. (A) Many studies that have examined the effect of dopamine on WM have participants use a binary match-to-sample paradigm. (B) Dopamine might modulate the representation of stored information in ways that cannot readily be detected using such methodology. For example, the resolution of the memoranda could be of varying quality or fidelity (fuzzier representations) but still be sufficient to provide a correct response, that is, a “yes” response in A could correspond to very different underlying representations. Binary report measures might fail to detect gradual changes in the fidelity of stored information with alterations in dopaminergic stimulation. Dopamine does not have to impact on WM in an all-or-nothing manner. (C) An alternative modulatory effect of dopamine might be on interference between the different memoranda, rather than on the quality with which their features are retained. In this scenario, the fidelity of a mental representation may be unaffected, but the features that make up the items may become confused (swapped) during the transition from perception to memory. For example, although the star was perceived as being orange, it is remembered as having the color of the other item (blue).

However, the impact that these subcortical filtering mechanisms have on the subsequent mental representations of memoranda remains to be determined. Previous studies that have attempted to resolve this question have employed binary report measures, for example, change detection tasks requiring a same/different judgment at retrieval (Figure 1A). These methods effectively probe WM to determine whether an item has been retained, whereas recent techniques that measure precision of recall have suggested an alternative view to this all-or-nothing, “quantal” account (reviewed in Ma et al., 2014). Furthermore, studies that have varied reward levels suggest that subtle differences can appear in the corruptibility of items in WM, an effect that may be dopaminergic in origin (Chumbley, Dolan, & Friston, 2008). This raises the possibility that D_2_ receptor stimulation may modulate distractor resistance at the subitem (i.e., feature) level and affect the resolution with which certain elements of those items are stored (Figure 1B). Alternatively, dopamine may have a role in altering interference between the items stored in memory, for example, on misbinding feature combinations belonging to different memoranda (Figure 1C). These two different sources of errors in WM can be teased apart using a probabilistic model of response selection (Bays et al., 2009; see also Figure 3A).

**Figure 2:**
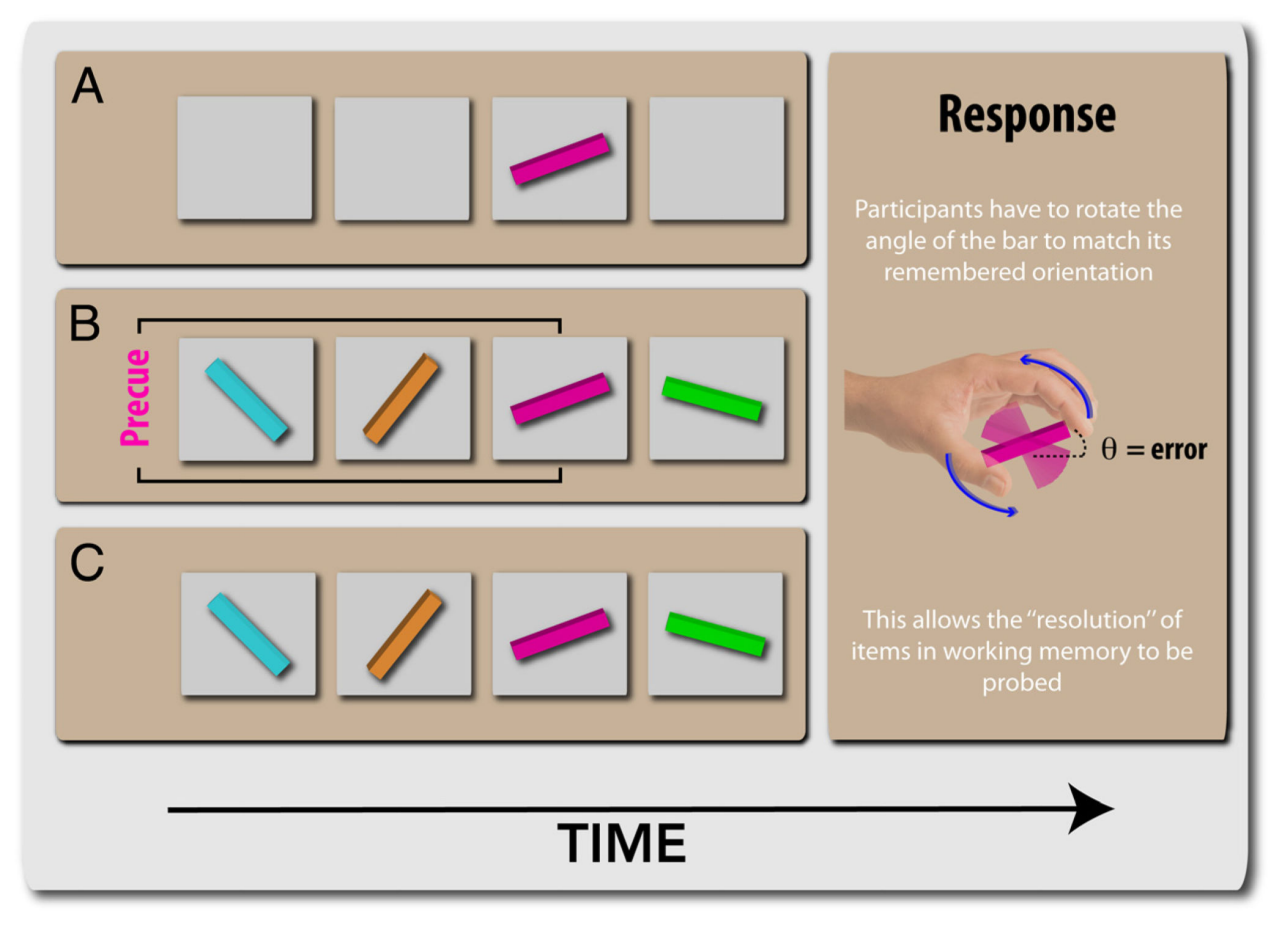
WM tasks. (A) One-item task: Participants had to maintain the orientation of one item for a variable duration of time before being probed to reproduce its orientation using a response dial, thereby providing a continuous measure of report on an analog scale. (B) Cued task: Participants were asked to retain only the orientation of the precued item (in this case, pink), which was the same color throughout a block. (C) Uncued task: Participants were asked to keep in mind all four oriented bars and were asked about one of these at response phase.

Here, we apply such a model to help understand how dopamine modulates information in WM. We examined the effect of a D_2_ agonist, cabergoline, on recall precision using a double-blind crossover, placebo-controlled design. We assessed recall performance under three different gating conditions: remembering only one item, being cued to remember only one target among distractors, and remembering all items (Figure 2). Crucially, we measured the quality of retained information by probing recall with an analog, continuous report scale (Gorgoraptis et al., 2011), rather than a binary one. This allowed us to both examine raw performance differences induced by cabergoline and also apply a probabilistic model to dissect out how different types of errors are affected by drugs.

## Methods

### Participants

Nineteen male participants were recruited to take part in the study (one provided incomplete data because of a computer error). Demographics are displayed in Table 1. Exclusion criteria were as follows: any current major illness, current or historical incident of psychiatric illness, and recreational drug use on more than one occasion in the past 6 months. Participants gave written informed consent, and the study was approved by the local ethics committee.

### Design

The study was a within-participant, double-blind placebo-controlled design. There were three sessions: baseline, first testing session, and second testing session. At baseline, participants were screened for drug contraindications, gave informed consent, and were familiarized with the paradigm. On the first and second testing sessions, they were administered 20 mg of domperidone (an antiemetic), followed 20 min later by either 1.5 mg of cabergoline or placebo (drug and placebo tablets were indistinguishable). A 1.5-mg dose was chosen so as to be greater than that given in a previous study where inconsistent effects on cognitive control were observed (1.25 mg; Frank & O’Reilly, 2006), with the addition of domperidone to mitigate potential physical side effects.

Cabergoline is a long-lasting D_2_ agonist (Andreotti et al., 1995). The tasks reported in this study were administered >2 hr after ingestion. For each test session, participants completed visual analog scales to measure mood, affect, physical side effects, and knowledge of the drug/placebo manipulation. Placebo/drug order was counterbalanced across participants. There was a minimum washout period of 2 weeks between the two test sessions.

### Tasks

The paradigm and related variants have been used elsewhere (Zokaei, McNeill, et al., 2014; Gorgoraptis et al., 2011). There were three conditions that differed according to whether distractor items were present and whether the targets/distractors were known or unknown (Figure 2). In the one-item task (Figure 2A), one colored bar was presented at screen center. Participants’ task was to remember the orientation of this colored bar. At the end of the trial, they were shown a probe colored bar, at a random orientation, with a circle around it to signal that this was the probe. Participants rotated the probe stimulus using a dial to match the orientation of the probed item to their memory. In this condition, there were four possible delays until the probe appeared.

The uncued and cued conditions were identical except that, in the cued condition, a 100% valid precue was presented, which indicated which of the four targets would be probed. For example, in Figure 2B, the precued item was pink. The same color was used as the precue throughout a block. By contrast, in the uncued condition, participants were not informed about which item they were going to be tested on and had to retain all four items. In both conditions, they were presented with four colored bars, displayed sequentially at screen center. The colors of the bars in each trial were selected randomly, with the condition that no color was repeated within a trial, from a set of five, easy-to-discriminate samples (red, yellow, green, blue, and purple). The orientations of the four bars were chosen from a flat distribution. In both conditions, items at each serial position were probed with equal frequency.

In the one-item condition, participants were shown only one colored bar, which appeared for 500 msec. To enable comparison with the other distractor-present conditions, the retention intervals were varied so as to match the four possible target probe durations that occurred when four items were presented. Thus, the delay periods between target offset and probe were 500, 1500, 2500, and 3500 msec. Note that, in the cued condition, distractors and targets would be distinguishable to the participant during encoding and only one item needed to be retained, similar to the one-item condition.

In each session (drug or placebo), there were 300 trials, split equally between the three conditions (one-item, cued, and uncued). Within in each condition, trials were split between each of the four serial positions (in the case of the cued and uncued conditions) or delay periods (one-item).

### Analysis

The metric of performance was angular error calculated as the absolute angular difference between target orientation and response orientation. The data were analyzed using a three-way repeated-measures ANOVA with SPSS 22.0 (IBM, Armonk, NY). Factors included Drug (placebo, cabergoline), Task (one-item, cued, and uncued), and Serial position (first, second, third, and fourth). Note that, in the one-item condition, as only a solitary item was present, Delay duration was used instead. Absolute angular error was log-transformed to render it suitable to linear analysis. WM capacity (digit span) and impulsivity as measured by the Barratt Impulsivity Scale (BIS; Patton, Stanford, & Barratt, 1995) have previously been used as proxies for striatal dopamine levels and have been found to predict the response to dopaminergic drugs (van der Schaaf, Fallon, ter Huurne, Buitelaar, & Cools, 2013; van Holstein et al., 2011; Cools, Gibbs, Miyakawa, Jagust, & D’Esposito, 2008; Cools, Sheridan, Jacobs, & D’Esposito, 2007; Kimberg & D’Esposito, 2003). Accordingly, one of the supplemental questions we addressed was whether digit span or impulsivity scores modulated the response to cabergoline.

### Modeling

Angular error gives us a measure of the fidelity of WM recall. However, it may be equally informative to examine how the pattern of errors changes with drug administration and condition. Thus, we fit a mixture model that dissociates different sources of error in memory (introduced by Bays et al., 2009; see also www.sobell.ion.ucl.ac.uk/pbays/code/JV10/). The mixture model is described by the following equation:

$$p\left(\hat{\theta }\right)=\alpha \phi_{\kappa }\left(\hat{\theta }-\theta \right)+\beta \frac{1}{m}\sum_{i}^{m}\phi_{\kappa }\left(\hat{\theta }-\varphi_{i}\right)+\gamma \frac{1}{2\pi }$$

**Figure 3:**
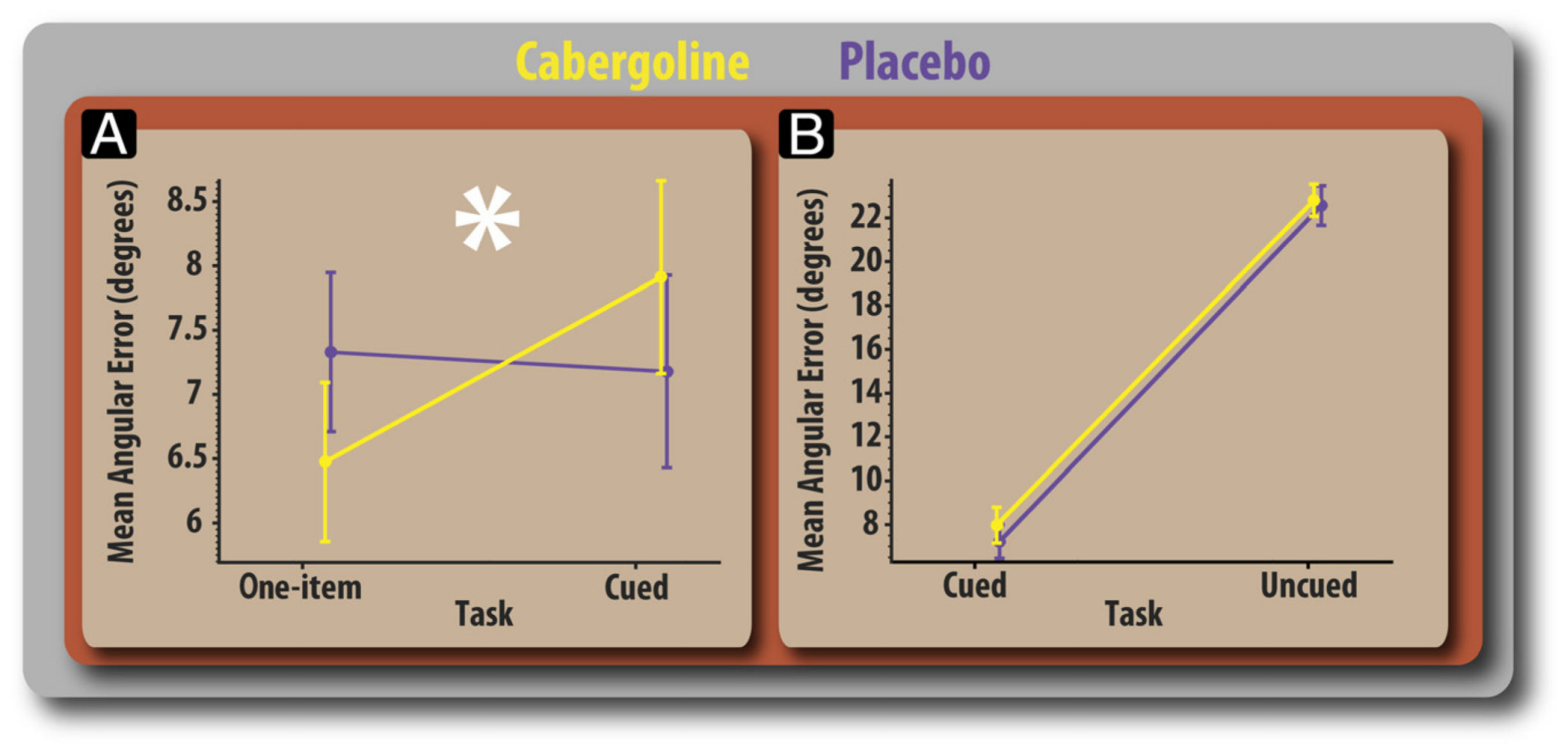
Absolute angular error. (A) Comparison of absolute mean angular error for the one-item and cued conditions split according to drug sessions. (B) Comparison of absolute mean angular error for cued and uncued conditions. Error bars reflect within-participant error (standard error of the difference between placebo and drug sessions).

This model partitions errors into four different components (Figure 3A):

1. Variability in precision (referred to as kappa [κ])
2. Probability of responding to the target orientation
3. Probability of responding to nontargets
4. Probability of guessing

Maximum-likelihood-derived parameters of κ, α, β, and γ were obtained using expectation maximization (Myung, 2003) produced for each participant (see Bays et al., 2009, for more details). It should be noted that the three parameters (α, β, and γ) are not independent as they must sum to 1.

We extracted the model parameters from each condition and drug session separately. Thus, there were 100 trials for each drug session and condition. Because of the number of trials, it was not possible to look at the effects of delay period.

### Model Evaluation

We evaluated whether the Bays et al. (2009) model was a good fit to the data in two ways. First, we evaluated whether the inclusion of the misbinding parameter (β) for the conditions with more than one possible target (cued and uncued) was redundant. We did this by comparing the model fit (Akaike Information Criterion [AIC]) for the cued and uncued conditions with and without the misbinding parameter. The model fit (AIC) for the full model (with misbinding) was 11702, and the AIC without the misbinding parameter was 11809. This led to a delta AIC of 107.19 in favor of the model that contained the misbinding parameter, suggesting that behavior was best explained in terms of misbinding.

As an additional indication of the goodness of fit, we compared the AIC for the model with and without the guessing parameter. The AIC for the full precision model (Bays et al., 2009) across all three conditions was 13135, whereas the AIC for the model without the guessing parameter was 13731, corresponding to an improvement of 33.1 log units per participant. Thus, the full model provided a better fit of the data compared with a model that did not contain the guessing parameter.

## Results

### Cabergoline-modulated Error according to Top–Down Attentional Demands

Cabergoline differentially affected recall error as a function of task, demonstrated by a significant Drug × Task interaction (*F*(2, 34) = 4.19, *p* = .024). The potentially multifaceted nature of this interaction necessitates that it is broken down into three separate analyses. The first considers how drug influences the effect that distractors have on performance (one-item vs. four-item cued conditions; Figure 3A). The second examines how cabergoline affects recall when multiple items were presented in the cued versus uncued conditions, each of which had four items in the sequence (Figure 3B). Finally, the third considers whether drug modulates the effect of load on recall (one-item vs. four-item uncued conditions).

**Figure 4:**
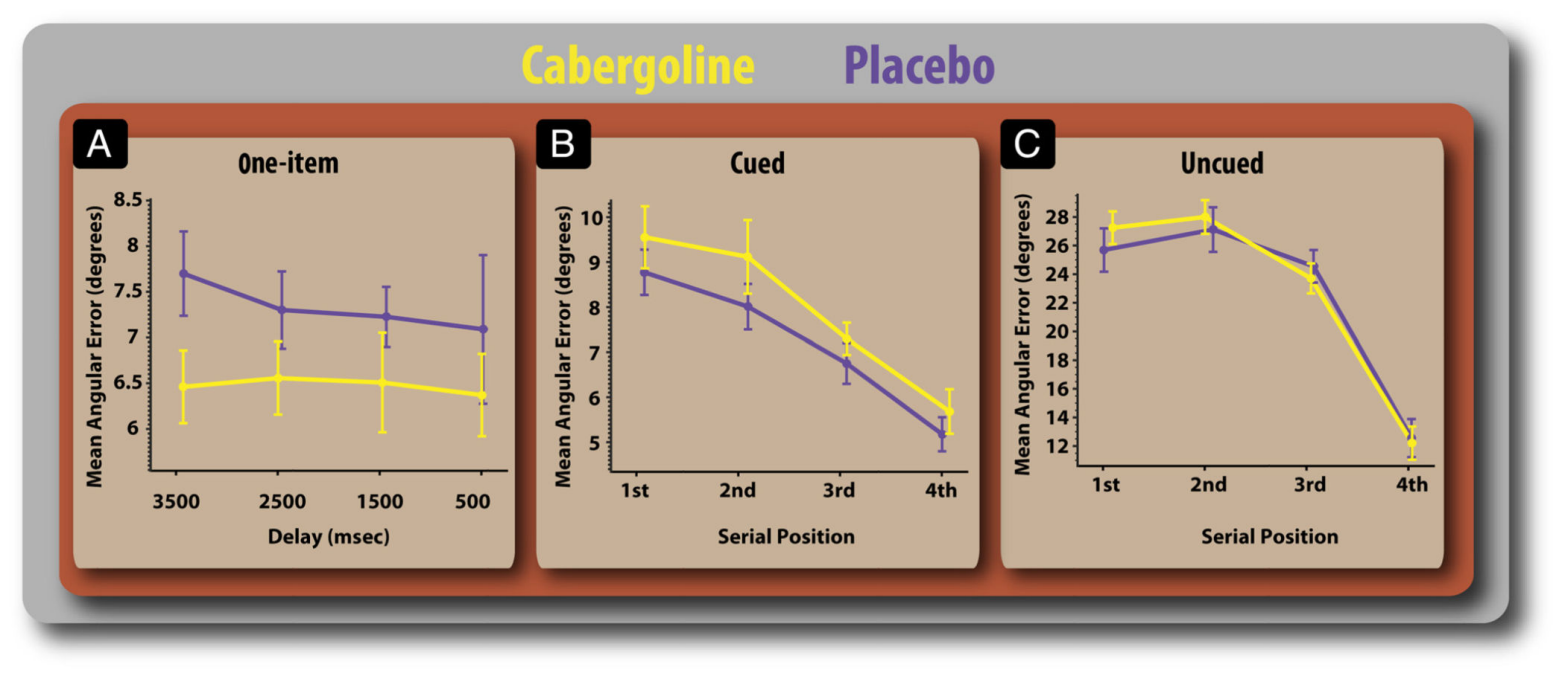
Details of performance in the three tasks on and off cabergoline. (A) Mean error according to retention delay in the one-item task. Performance according to serial position of probed item in the precued (B) and uncued (C) tasks. There was no interaction between drug and delay duration or serial position. Error bars reflect *SEM*.

Comparison of recall error (in degrees) for the one-item versus four-item cued conditions revealed that cabergoline significantly modulated the effect that distractors had on report (*F*(1, 17) = 12.69, *p* = .002). On placebo, participants showed no significant difference in performance between the two tasks (*t*(17) = 0.67, *p* = .521). In line with previous data (Gorgoraptis et al., 2011), this demonstrates that people are able to filter distractors very effectively. After cabergoline, however, recall error was significantly lower (1.45° on average) for the one-item compared with the four-item cued condition (*t*(17) = 2.61, *p* = .018). Thus, cabergoline improved performance when one item had to be retained but worsened it in the four-item precued condition (Figure 3A). Enhanced performance on cabergoline in the one-item task was observed at all delays (Figure 4A).

Next, we examined whether cabergoline affected recall error when multiple (four) items were presented in the cued versus uncued conditions. Although precueing clearly improved performance overall, there were no significant effect of drug in the comparison between cued and uncued tasks (*F*(1, 17) = 1.139, *p* = .30; Figure 3B) and no evidence that cabergoline affected recall according to load (one-item vs. four-item uncued; *F*(1, 17) = 0.310, *p* = .585).

Thus, the only effect of cabergoline was observed in the condition where one item presented alone had to be remembered (performance enhanced) versus the task in which one item was precued with 100% validity in a sequence of four items (performance deteriorated). Recall performance improved on the drug when there were no distractors but worsened when distractors had to be gated out in the cued condition. This was not affected by memory retention interval (Figure 4), indicating that cabergoline did not alter decay of items stored in WM.

Aside from the Drug × Task interaction, there were no significant main effect of Drug on error and no significant interaction between Drug and Delay (*F*s < 1). Other non-drug-related effects were comparable with those observed on this task previously (Gorgoraptis et al., 2011): Error was greatly influenced by Task (*F*(2, 34) = 215.59, *p* < .0001), and Serial position had a significant effect on error (Figure 4A–C; *F*(3, 51) = 61.36, *p* < .0001), such that later items were recalled more accurately—the classical recency effect. Serial position had greater effects on error in the uncued condition (Figure 4C; *F*(6, 102) = 24.73, *p* < .0001). However, drug did not significantly affect this relationship (*F* < 1).

### Cabergoline Modulates the Fidelity of Recall When Distractors Were Present

**Figure 5:**
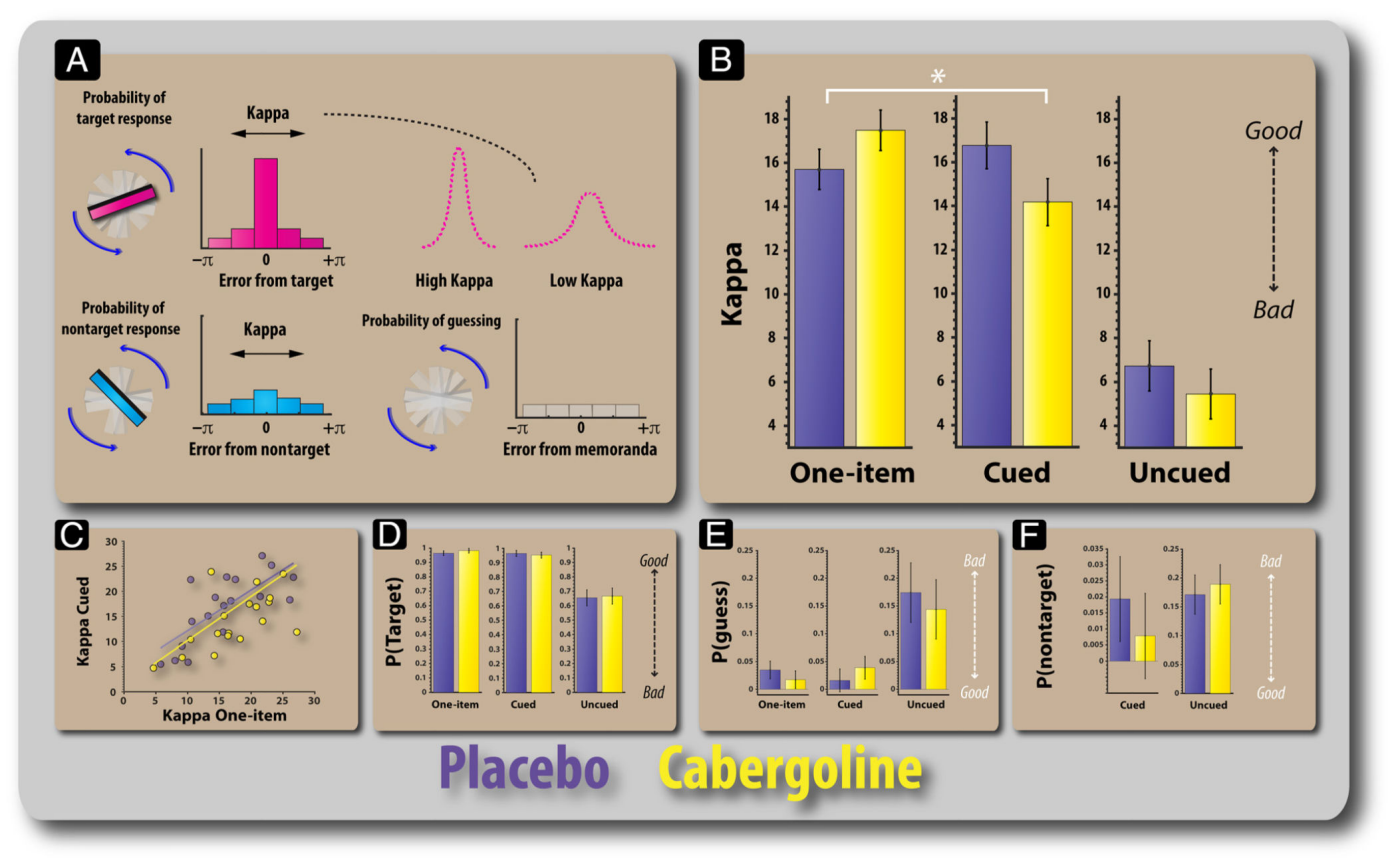
Modeling results for the placebo and cabergoline sessions. (A) Error can arise in recall because of increased variability in remembering the orientations of the probed item, which is captured in the model in terms of the parameter (kappa). A high kappa corresponds to lower variability in the precision of retained items (for targets or nontargets). Error is also expected to arise through random guessing/responses. Performance on the task not only requires an ability to accurately remember the orientations of bars but also the ability to bind, or associate, an orientation to a specific color. Thus, errors could arise through misbinding remembered orientations with remembered colors. For example, if the pink bar appeared at an orientation of 40° and the cyan bar appeared at 135°, a misbinding error would be said to have occurred if they rotate the probed pink bar to the remembered orientation of the cyan bar. (B) Kappa values according to task and drug. There was a significant difference in kappa on the cued condition and a trend for the one-item condition. (C) Plot showing the relationship between the kappa values for the one-item and precued conditions. (D) Probability of responding to the target (probed item) according to task and drug session. (E) Probability of random guessing according to drug and task. (F) Probability of responding to the distractor (cued task) or nontarget (uncued task) orientation. Error bars reflect the standard error of the difference between placebo and drug sessions.

Given that the differences in angular error according to drug and task reported previously might be due to several factors (reduced precision, reduced responding to target, responding to nontarget orientations, or increased guessing), we applied a probabilistic model of response selection (Bays et al., 2009) to tease these components apart (Figure 5A).

First, we examined kappa, which is the modeled concentration of the response around the target item—a measure of the fidelity of the item representation in WM. A high kappa indicates a high concentration around the target item, whereas a low kappa corresponds to a wider distribution or greater variability. Kappa values were significantly modulated by drug and task (*F*(1.5, 25.2) = 4.66, *p* = .028; Figure 5B and C). As for raw angular error, this was driven by a differential effect of cabergoline in the one-item versus cued conditions (*F*(1, 17) = 17.54, *p* = .001), with a significant difference on the two tasks appearing only on drug (*t*(17) = 2.72, *p* = .014). Moreover, there was a trend toward higher kappa under cabergoline compared with placebo in the one-item task (*t*(17) = 1.933, *p* = .07). In the cued condition, kappa was significantly lower on cabergoline compared with placebo (*t*(17) = 2.42, *p* = .027).

For misbinding (cued and uncued), there was no significant interaction between drug and task (*F* < 1; Figure 5E). Thus, there was no evidence that cabergoline led to increased confusion of the feature dimensions of memoranda or interference between stored memoranda. There were no significant differences between drug sessions in the probability of responding to the target (probed item; Figure 5D) or in chance responding (guessing; Figure 5E). Thus, the difference in error rate according to drug in the cued condition appears to be due to reduced precision of the items stored in WM. In accordance with the lack of an effect of cabergoline on performance on the uncued condition, there was no significant effect of drug on kappa, probability of responding to the target and nontargets, or guess rate.

### Supplemental Analysis

A supplementary question concerns the role of individual differences in modulating the abovementioned effects of cabergoline on precision (kappa). To this end, we examined the role of digit span and impulsivity (BIS score) in influencing the effects of drug and task by entering these values as *z* scored covariates separately into our repeated-measures ANOVA. Digit span was not found to modulate the effect of cabergoline on overall precision (*F* < 1) or influence, in a three-way manner, the interaction between Drug and Task (*F* < 1). Similarly, total impulsivity (BIS score) did not influence the effect of drug or the interaction between Drug and Task (*F*s < 1).

## Discussion

Gating entry of information into WM is an essential function that enables humans to perform complex tasks (D’Esposito & Postle, 2015; Gazzaley & Nobre, 2012; Braver & Cohen, 2000). Effective gating is important, given the limited capacity of WM (Fallon, Zokaei, & Husain, 2016), and it has been proposed that dopamine plays a crucial modulatory role in this process (Hazy et al., 2007; Frank & O’Reilly, 2006). The present results establish the importance of the dopaminergic system in facilitating gating under varying levels of top–down control. In this study, a D_2_ agonist, cabergoline, was able to modulate the resolution with which information held in WM is reported. Cabergoline affected recall performance for a single item differentially, depending on the presence of distractors during encoding. Whereas cabergoline improved recall in the absence of distractors, it worsened recall when distractors had to be gated out in the cued condition (Figure 3A and B). This was not affected by the memory retention interval (Figure 4A–C), indicating that cabergoline did not alter the decay of items in WM.

The differential effects of drug on error in the one-item and cued conditions appeared to result from changes in the quality or fidelity with which relevant items were represented in memory (Figure 5A). There was no evidence that cabergoline altered the extent to which participants became confused between targets and nontargets, that is, there was no increase in incorrectly conjoining a color and an orientation—interference (Figure 5F). Taken together, the results show that cabergoline does not appear to have a generic enhancing or deleterious effect on WM. Rather, its effects can be predicted on the basis of the need to exert top–down attentional control about what information to encode into memory.

Dopamine has a long association with WM, either through acting at the level of the frontal cortex (Smith, Swift-Scanlan, & Boettiger, 2014; Vijayraghavan et al., 2007; Abi-Dargham et al., 2002; Brozoski, Brown, Rosvold, & Goldman, 1979) or the striatum (Bäckman et al., 2011; Braskie et al., 2011; Clatworthy et al., 2009; Cools et al., 2008; Brozoski et al., 1979). It has also been implicated in aspects of attentional control (Soltani, Noudoost, & Moore, 2013; Noudoost & Moore, 2011; Chudasama & Robbins, 2004; Crofts et al., 2001; Servan-Schreiber, Carter, Bruno, & Cohen, 1998). These twin and often intermingled effects on selective attention and WM have been reconciled within computational models and supported by empirical findings that have posited an antagonistic relationship between stability and flexibility (Fallon & Cools, 2014; Fallon, van der Schaaf, ter Huurne, & Cools, 2017; Cools & D’Esposito, 2011; Colzato, Waszak, Nieuwenhuis, Posthuma, & Hommel, 2010; Durstewitz & Seamans, 2008; Hazy et al., 2007; Müller et al., 2007; Nolan, Bilder, Lachman, & Volavka, 2004). These reports argue that improving stability may also manifest itself as impaired flexibility.

From the findings of this study, it could be argued that cabergoline—a D_2_ agonist—acts to impair selective attention to task-relevant stimuli but improve WM maintenance. Specifically, in the absence of distractors (pure maintenance in the one-item condition), recall precision was relatively enhanced under conditions of heightened D_2_ stimulation. In contrast, in the presence of “known distractors” (cued condition), cabergoline actually impaired WM performance, by worsening precision. This finding is particularly congruent with influential theories, which claim that WM gating occurs through modulation of the balance between activity of the direct (go) and indirect (no-go) pathways that link the striatum to cortex (Hazy et al., 2007; Frank & O’Reilly, 2006). These models have sought to explain how and why certain features are allowed to enter WM—a function often ascribed to the “central executive” (Baddeley, 2012). In the context of WM, this is achieved through a division of labor with activation of the go pathway permitting current perceptual stimuli to enter prefrontal-mediated mnemonic networks and activation of the no-go pathway preventing current perceptual information from entering these networks.

On the basis of the Frank and O’Reilly (2006) model, it might be argued that the effects reported in our study occur because of the postsynaptic effects of D_2_ stimulation. Postsynaptic D_2_ receptors exert a generally inhibitory action on neurons, through actions on adenylyl cyclase (Nicola, Surmeier, & Malenka, 2000). Within the Frank and O’Reilly (2006) model, cabergoline, when acting postsynaptically, is viewed as inhibiting no-go pathway activity, namely, in removing the net inhibitory output of the external pallidum and subthalamic nucleus. This inhibition of the no-go pathway may lead to a preponderance of activity in the go pathway. For example, under cabergoline, the go, that is, encode, signals from the BG that accompany the appearance of items on the screen may be enhanced irrespective of top–down goals and expectations.

Enhanced, indiscriminate generation of go signals could explain the present results. In the one-item condition, augmentation of go signals may enhance the quality of the representation of the item to be retained, whereas the same neurophysiological response may have deleterious consequences in the cued condition, where go response to distractors needs to be suppressed. Signals emanating from the BG have been shown to be endowed with this capacity. It has already been shown in the domain of attention that signals in the BG can enhance activity in task-relevant areas of the cortex and diminish activity in task-irrelevant areas. Neurophysiologically, these changes may correspond to changes in neuronal oscillations at the alpha (10 Hz) frequency, which are thought to index functional inhibition (Jensen & Mazaheri, 2010) and have previously been found to relate to the precision with which items are stored in WM (Myers, Stokes, Walther, & Nobre, 2014) and distractor resistance (Bonnefond & Jensen, 2012). Thus, stimulation of postsynaptic D_2_ receptors with cabergoline, which inhibit no-go activity, could lead to reduced inhibition of taskirrelevant processing areas, that is, a failure in functional inhibition. This could correspond to increased distractability, which is potentially congruent from findings with Parkinson’s disease where the administration of dopaminergic agents can increase distractability (Georgiev et al., 2015; Cools, Miyakawa, Sheridan, & D’Esposito, 2010).

One finding that may appear puzzling is cabergoline’s lack of effect on performance in the uncued task (Figures 3B and 4C). However, this may have occurred not because cabergoline has no effect on recall in this condition but because the end result of D_2_ stimulation is to produce the same effects as is normally the case, that is, placebo. There is a wealth of evidence to suggest that items that are presented more recently are recalled with a higher fidelity (Gorgoraptis et al., 2011) and that the most recent (last) item presented may have a special—privileged—state in WM (Cowan, 2011; Oberauer, 2002), leading it to be recalled with greater accuracy. In Figure 4C, we see that there are strong serial position effects in both placebo and cabergoline conditions for the uncued task. However, the mechanisms underlying this serial position effect may be different. Under placebo, the most recently presented item may be recalled with greater acuity because of the normative reasons through which recent items are recalled better (Gorgoraptis et al., 2011). In contrast, under cabergoline, the effect of D_2_ stimulation—indiscriminate activation of go signals in the BG—may serve to unwittingly produce the same effect. For example, during the uncued condition in which the stimuli are consecutively appearing, the most recently presented item may keep getting assigned the lion’s share of mnemonic resources, because of the D_2_ effects, ultimately producing a scenario where the last (most recent) items are remembered with greater fidelity. However, because this is also the pattern that occurs in the normative situation (on placebo), no drug effects are observed. As such, the uncued task is unable to discriminate between cabergoline and placebo. It should be noted, though, that this explanation is speculative. However, the issue could be resolved empirically by using a retrocue design, whereby participants are informed, after encoding, which item they are going to be tested on. These designs have proven effective in uncovering the mnemonic and neural basis of resource allocation shifts during WM trials (Myers, Walther, Wallis, Stokes, & Nobre, 2015; Zokaei, Manohar, et al., 2014). Future studies should use such designs to unmask the effect of dopamine on WM resource allocation.

The current study has provided support for the idea that dopamine can affect the precision with which mental representations are formed and acted upon in the mind. This demonstrates that dopamine can have a graded effect in influencing the fidelity of recall, rather than the binary effect that is often tacitly assumed. It should be noted however that, based solely on the present work, we are unable to specify where—in the processes between perception and storage—this graded effect originates. This is important as, although dopamine’s effect on WM representations may be graded, this may have been produced by the existence of a binary effect at some other cognitive subsystem. For example, the effect of cabergoline on precision in the cued task may have been caused by a binary (all-or-none) effect on the probability of updating an item into WM (irrespective of whether they are targets or distractors). In this scenario, there could be a lower threshold for updating items into WM, meaning that there are more items fighting for mnemonic resources, which would produce the reduced precision that was observed here. We cannot conclusively rule out that such mechanisms were responsible for producing the present results. However, such an explanation seems incapable of accommodating all of the results as, even in the one-item condition where there is no competition for resources, cabergoline was still seen to affect recall. Thus, dopaminergic stimulation does appear to affect the fidelity of mental representations.

It is also possible that whether dopamine has a binary or graded effect on mental representations may vary according to what neural loci dopamine is affecting. One computational rationale for including the BG in WM processes is that it allows mental representations to be selectively updated or removed in concert with temporally precise reward signals in the striatum (Fallon & Cools, 2014; Chatham & Badre, 2013; O’Reilly & Frank, 2006). In contrast, dopaminergic stimulation of the pFC could have an all-or-none effect on mental representations by totally destabilizing—collapsing—the prefrontal circuits responsible for maintaining whole items, similar to what occurs under stress (Arnsten, 2007). Thus, the precise interplay between the frontal cortex and BG may determine how binary or graded dopamine’s effects may be.

Despite being congruent with prior research suggesting that D_2_ stimulation can modulate distractor resistance, a novel finding from this experiment is that changes in recall performance occur in the presence of distractors, crucially without any corresponding change in the probability of misbinding or interference between items. Misbinding events occur when different stimuli features are inappropriately combined together. In the present context, this would correspond to pairing an orientation with the wrong color. Cabergoline did not significantly affect misbinding rates on any of the tasks used here. Thus, D_2_ agonism did not cause any confusion of relevant and irrelevant items—a possibility that previous studies have not been able to authoritatively rule out (Figure 1C). This suggests that D_2_ receptors do not modulate interference between items in WM and, conversely, that augmenting dopamine levels may have little impact on treating cognitive impairment where such confusion between items is prominent. Increased misbinding has, for example, been associated with damage to the hippocampus (Liang et al., 2016; Pertzov, Miller, et al., 2013), indicating that the medial-temporal lobe may be important in correctly combining feature dimensions. It could be argued, however, that our study is not in a place to make this conclusion because the task, particularly the cued condition, was too easy as no misbinding occurred (Figure 5F). Arguing against this, however, is the fact that drug effects on misbinding were not found in the uncued condition, although this task is considerably harder and does induce substantial misbinding (Figure 5F). Moreover, if cabergoline were to have had severe effects on selective attention, then performance on the cued condition would start to resemble the uncued condition, in that the two conditions were identical except for the fact that participants were told in the precue condition what item they are subsequently going to be probed on. Thus, there was ample room for participant’s performance to degrade if cabergoline did indeed induce misbinding. The fact that cabergoline did not increase misbinding in this condition, however, suggests that the memory-enhancing effect of the precue is not influenced or perturbed by cabergoline. In other words, were cabergoline to have increased misbinding (confusing targets with nontargets), then performance would not have been at ceiling.

The sample size in this study is relatively small. One concern is that the results could be a false positive. However, this is unlikely to be the case. The results of this study are highly congruent with findings from several previous investigations that have articulated a role for dopamine, specifically D_2_ stimulation, in modifying distractor resistance (Bloemendaal et al., 2015; Cools et al., 2007; Mehta et al., 2004). They also generally conform to the predictions generated by computational models (Frank & O’Reilly, 2006). The findings of this study build on previous work by specifying the effect that distractor suppression has on the underlying mental representation of relevant information.

In summary, this study has revealed that dopaminergic D_2_ agonists can affect the resolution of items in WM, even when only one item needs to be maintained. However, the effect of cabergoline on mnemonic representations appeared to depend greatly on the need to gate the entry of information into WM. There was no effect of cabergoline on WM representations when multiple items needed to be remembered and no gating was required. In contrast, cabergoline impaired mnemonic representations of items encoded selectively.

## Figures and Tables

**Table 1:** Participant Demographics

*Metric*	*Mean*	*SD*	*Min*–*Max*
Age, Years	26.6	5.8	18–36
Years of education	10.2	2.1	5–14
Raven’s advanced progressive matrices	7	2.8	1–11
Digit Span Forward	11.8	2.1	8–14
Barratt Impulsivity Scale	67.2	10.7	47–84

Years of education refer to the number of years since leaving U.K. primary school (~11 years).
